# Identification of TLE Focus from EEG Signals by Using Deep Learning Approach

**DOI:** 10.3390/diagnostics13132261

**Published:** 2023-07-04

**Authors:** Cansel Ficici, Ziya Telatar, Onur Kocak, Osman Erogul

**Affiliations:** 1 Department of Electrical and Electronics Engineering, Ankara University, 06830 Ankara, Turkey; 2 Department of Biomedical Engineering, Başkent University, 06790 Ankara, Turkey; ztelatar@baskent.edu.tr (Z.T.); okocak@baskent.edu.tr (O.K.); 3 Department of Biomedical Engineering, TOBB University of Economics and Technology, 06560 Ankara, Turkey; erogul@etu.edu.tr

**Keywords:** EEG, temporal lobe epilepsy, deep learning, epileptic focus detection

## Abstract

Temporal lobe epilepsy, a neurological disease that causes seizures as a result of excessive neural activities in the brain, is the most common type of focal seizure, accounting for 30–35% of all epilepsies. Detection of epilepsy and localization of epileptic focus are essential for treatment planning and epilepsy surgery. Currently, epileptic focus is decided by expert physician by examining the EEG records and determining EEG channel where epileptic patterns begins and continues intensely during seizure. Examination of long EEG recordings is very time-consuming process, requires attention and decision can vary depending on physician. In this study, to assist physicians in detecting epileptic focus side from EEG recordings, a novel deep learning-based computer-aided diagnosis system is presented. In the proposed framework, ictal epochs are detected using long short-term memory network fed with EEG subband features obtained by discrete wavelet transform, and then, epileptic focus identification is realized by using asymmetry score. This algorithm was tested on EEG database obtained from the Ankara University hospital. Experimental results showed ictal and interictal epochs were classified with accuracy of 86.84%, sensitivity of 86.96% and specificity of 89.68% on Ankara University hospital dataset, and 96.67% success rate was obtained on Bonn EEG dataset. In addition, epileptic focus was identified with accuracy of 96.10%, sensitivity of 100% and specificity of 93.80% by using the proposed deep learning-based algorithm and university hospital dataset. These results showed that proposed method can be used properly in clinical applications, epilepsy treatment and surgical planning as a medical decision support system.

## 1. Introduction

Temporal lobe epilepsy is the most common type of epilepsy, characterized by high-amplitude and rhythmic EEG signal patterns suddenly arise from a specific brain area, defined as epileptic focus, and then mostly generalized to whole brain. Considering that EEG signal patterns of epilepsy patients are different from healthy individuals and, diagnosis of epilepsy can be conducted by an expert examining long-term EEG recordings together with clinical findings. Drug-resistant TLE cases can be treated with surgery, so TLE focus localization is crucial for treatment planning. 

In the literature, some approaches exist about epilepsy detection from EEG signals. Current studies mainly focused on classifying ictal and interictal epochs by using various machine learning [1,2,3,4,5,6,7,8,9,10,11,12,13,14,15] and deep learning methods [16,17,18,19,20,21,22,23,24,25,26,27,28,29]. 

As mentioned in [30], SVM is faster training phase speed than deep learning-based algorithms, and it is one of the preferred classifiers among others like KNN and LDA. Most of the studies with SVM classifier in the literature used Bonn EEG dataset [31], which includes intracranial cleared EEG recordings with higher classification accuracies. Bhattacharyya et al. [1] classify EEG signals in order to detect epilepsy by using tunable-Q wavelet transform-based multiscale entropy measure. They used Bonn University database that have cleared five EEG sets from a single channel and reported that they achieved accuracy of 98.60% in discriminating seizure and non-seizure classes. Ficici et al. [2] classified seizure from Bonn University EEG dataset by using SVM method with accuracy of 99.00%. Zarei et al. [3] introduced a method with DWT and OMP techniques to detect epileptic seizures by using SVM. Khan et al. [4] classified EEG signals as epileptic and non-epileptic by examining discriminant analysis based on local binary pattern and statistical features of DWT with accuracy of 99.60% on CHB-MIT dataset. Raghu et al. [5] examined a method detecting epilepsy by using SVM with sigmoid entropy of EEG subbands. They obtained 100% and 94.21% sensitivities on Bonn University and CHB-MIT databases, respectively. Chen et al. [6] employed SVM with RBF method to discriminate ictal and interictal EEG segments using DWT-based statistical signal features from Bonn University dataset with accuracy of 99.30%. Richhariya et al. [7] introduced a method for distinguishing seizure and seizure-free intervals by using USVM from Bonn University dataset with accuracy of 99.00%. Sharma et al. [8] detected ictal EEG signals by using SVM applying on TUH-EEG dataset, and they obtained 79.34% success rate.

Qureshi et al. [9] used KNN and FRNN methods for classification of epileptic seizure from EEG signals. They applied their algorithms to Bonn and CHB-MIT databases and obtained 99.81% and 92.79% accuracies, respectively. Mursalin et al. [10] presented an automated epileptic seizure detection method using random forest classifier. They obtained highest accuracy of 99.00% on Bonn dataset. Omidvar et al. [11] proposed epilepsy seizure detection approach using five-level Daubechies (Db4) DWT and ANN-SVM classifiers. They obtained accuracies of 100% for two-class classification and 98.70% for three-class classification on Bonn dataset. Acharya et al. [12] detected epileptic seizure by attempting GMM, WPD and PCA. They achieved 99.00% success rate in three-class classification on Bonn dataset. Ficici et al. [13] proposed a machine learning-based method to discriminate TLE patient, PNES patient and healthy subjects. They obtained interictal and ictal classification accuracy of 98.00% on Bonn and CHB-MIT databases using single channel EEG. Li et al. [14] performed three-class classification on Bonn dataset by using neural network ensemble method with accuracy of 98.80%. Slimen et al. [15] conducted interictal and ictal epoch classification by using SVM, KNN and LDA on CHB-MIT dataset with accuracy of 100%. 

Recently, the following noticeable advantages have been achieved with deep learning-based algorithms applied on biomedical signals in comparison with the SVM-based classifiers: (1) Deep learning has potential for multi-class identification and may achieve more accurate results in comparison with SVM when it modified as a multi-classifier; (2) Deep learning has higher potential for handling large-scale data classification than the SVM-based algorithms [30].

Qiu et al. [16] presented a method for epileptic seizure detection by examining ResNet LSTM network on Bonn University dataset, and they achieved accuracy of 99.78% for interictal and ictal classification. Fraiwan et al. [17] classified the EEG data from single channel EEG as focal and non-focal by attempting LSTM with accuracy of 99.20%. Poorani et al. [18] proposed a deep learning-based epilepsy detection method from EEG signals. They applied CNN and LSTM methods on CHB-MIT database and obtained a classification accuracy of 94.83%. They obtained 100% and 97.10% success rates on Bonn University and CHB-MIT databases, respectively. Varlı et al. [19] proposed an approach to classify multiple EEG signals by applying CWT, STFT and LSTM. They performed their algorithms on three different publicly available datasets which are CHB-MIT, Bern-Barcelona and Bonn University EEG records and obtained highest accuracy of 99.62% in interictal and ictal discrimination on Bonn University dataset. Daoud et al. [20] proposed a deep learning approach for epileptic focus localization and implemented on FPGA. They obtained accuracies of 93.21% and 96.00% on Bern-Barcelona and Bonn datasets, respectively. Mir et al. [21] presented deep learning-based framework with LSTM for diagnosis of epileptic seizure from EEG and achieved 99.80% accuracy on CHB-MIT dataset. Singh et al. [22] predicted epileptic seizures by using LSTM on CHB-MIT dataset with classification accuracy of 98.14%.

Yildiz et al. [23] applied CNN classifier to classify three-class dataset of Bonn University and achieved 100% success rate. Türk et al. [24] proposed scalogram-based CNN to detect epilepsy from EEG signals. They obtained success rate of 98.50% in interictal and ictal classification, and 80.00% in epileptic focus detection by using Bonn University cleared EEG database. Lebal et al. [25] used CNN and RNN for epilepsy detection using one-channel and multichannel EEG signals applied on Bonn and CHB-MIT datasets and achieved 100% and 98.22% success rates, respectively. Ilias et al. [26] classify EEG signals to detect epilepsy by using STFT and CNN methods. They obtained 97.00% success rate in separating based on three-class the Bonn dataset. Gao et al. [27] introduced a deep CNN method to classify ictal and interictal intervals obtained from CHB-MIT database with accuracy of 92.60%. Farooq et al. [28] proposed a survey showing the taxonomy studies about epilepsy detection in the literature. Islam et al. [29] introduced a seizure detection survey from EEG. 

This paper introduces a retrospective study using multichannel EEG signals recorded from TLE patients. In the proposed approach, an LSTM network was applied to detect seizure first, and then localization of epileptic focus region, in left or right brain hemispheres, and was examined by handling EEG channel energy asymmetry. Although, there are lots of study on epilepsy in the literature, most of them focused on only ictal and interictal classification instead of epileptic focus localization using known cleared databases. Novelty of this study is given as detecting seizure intervals and identifying epileptic focus sequentially by using EEG signals. The proposed method can also be used properly in clinical applications, epilepsy treatment and surgical planning as a medical decision support system.

Rest of the paper is organized as follows: In Section 2, datasets and methods used in this study are described in detail. In Section 3, experimental results and comparison with previous studies are reported. Analysis and discussions are presented in Section 4. Also, limitations, future works and conclusions of the proposed study are reported in Section 4.

## 2. Materials and Methods

**Figure 1:**
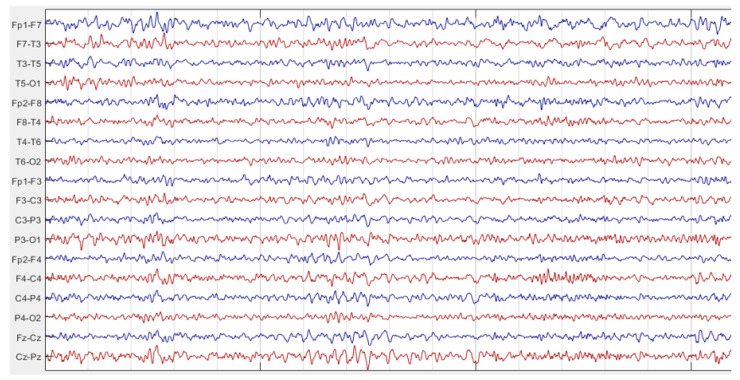
EEG recording sample of interictal interval.

**Figure 2:**
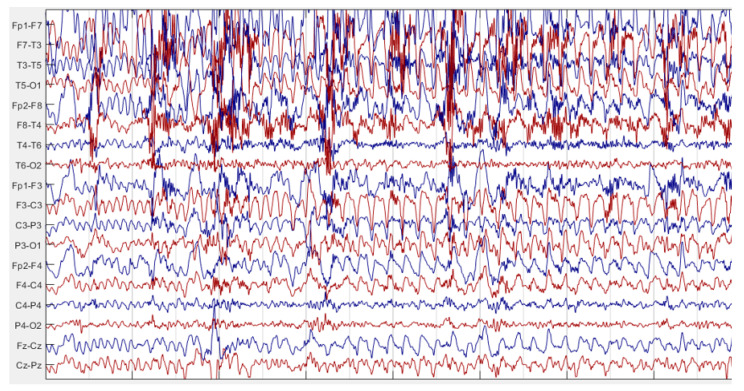
EEG recording sample of ictal interval.

In this study, multichannel EEG recordings of TLE patients obtained from the Ankara University hospital and Bonn University dataset were used. Scalp EEG signals of AU dataset were sampled at 500 Hz and recorded using 18 channels obtained by bipolar 10–20 electrode placement. EEG signals recorded from Fp1-F7, F7-T3, T3-T5, T5-O1, Fp2-F8, F8-T4, T4-T6, T6-O2, Fp1-F3, F3-C3, C3-P3, P3-O1, Fp2-F4, F4-C4, C4-P4, P4-O2, FZ-CZ, CZ-PZ channels. This database includes EEG recordings of 27 TLE patients. Figure 1 and Figure 2 are the sample intervals of original recordings pointing out interictal and ictal periods. These figures were plotted by using Matlab [32]. Ictal and interictal EEG intervals were labeled retrospectively by the expert. In addition, whole EEG recordings of patients were labeled as left TLE or right TLE by the expert. Rhythmic theta and beta activities, repetitive spikes, sharp waves and low-voltage rapid activity were the ictal period indicators of TLE for the neurologist marking on EEG recordings. So, the labelling by expert was regarded as gold standard for the performance evaluation of this study. This algorithm was implemented with MATLAB 2021a [33] via the computer, Intel Core i7, 2.60 GHz processor with 16.0 GB RAM.

**Figure 3:**
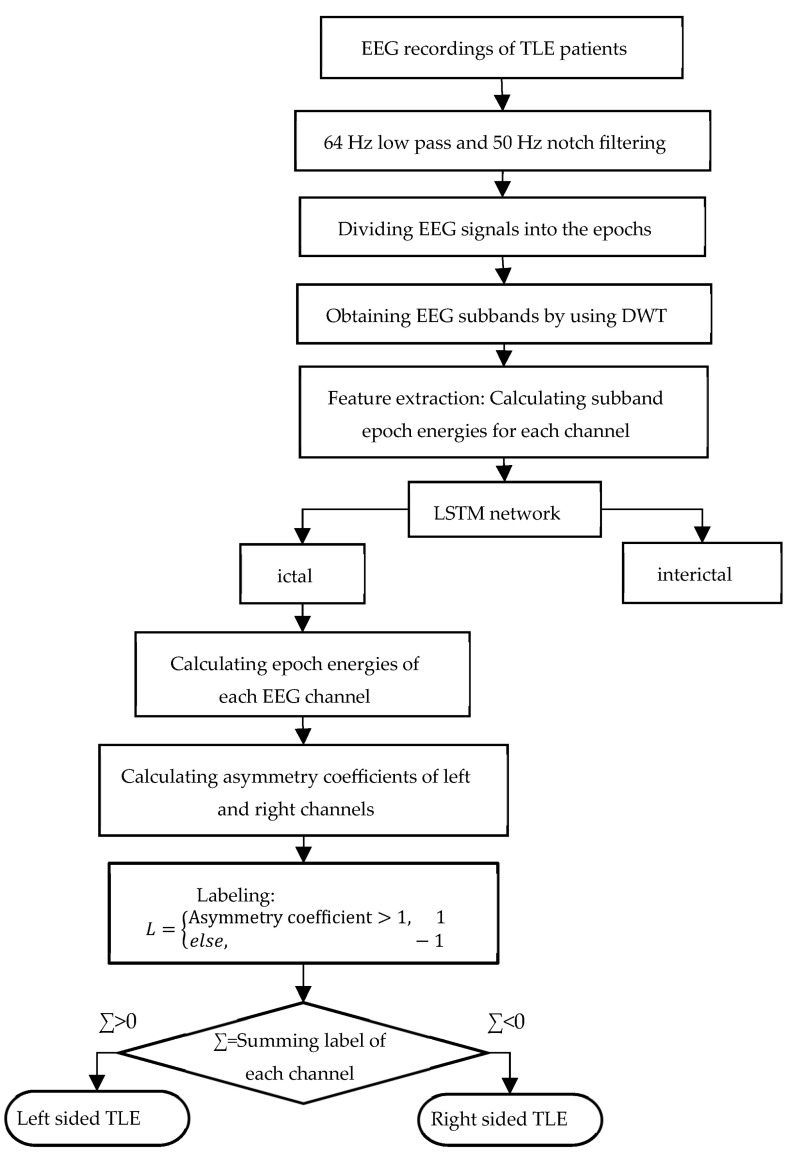
Flowchart of the epileptic focus identification algorithm.

In the proposed algorithm, initially ictal EEG intervals are detected for identification of epileptic focus from ictal EEG signals. On the other hand, the method is composed of two main parts; first one is detection of ictal epochs and second one is identification of epileptic focus. A whole flowchart indicating the proposed epileptic focus localization algorithm is shown in Figure 3. In ictal epoch detection part, firstly, EEG recordings of TLE patients are filtered by Butterworth-type low-pass filter with cutoff frequency of 64 Hz to obtain desired frequency range for DWT and 50 Hz notch filter to remove power line noise. Secondly, EEG signals are segmented into periods of 4096 samples called as epoch. Thirdly, DWT decomposition method is applied to obtain EEG signal subbands. Fourthly, EEG signal features, as the inputs of deep learning network, are extracted by calculating subband epoch energies of each EEG channel. Fifthly, ictal and interictal epochs are classified by using LSTM network. In the epileptic focus identification part, initially, energies of ictal EEG signals, detected in the first part, are calculated for each channel. Then, asymmetry coefficients are calculated from left and right symmetric EEG channels mentioned in Section 2.1. 

### 2.1. Feature Extraction

In this study, LSTM classifier was fed with extracted signal features obtained from raw EEG data. To obtain signal subbands, nine-level discrete wavelet decomposition was used with fourth-order Daubechies (db4) wavelet function. Thus, 10 signal subbands that are D1 (32–64 Hz), D2 (16–32 Hz), D3 (8–16 Hz), D4 (4–8 Hz), D5 (2–4 Hz), D6 (1–2 Hz), D7 (0.5–1 Hz), D8 (0.25–0.5 Hz), D9 (0.125–0.25 Hz) and A9 (0–0.125 Hz) were obtained. Then, single-level DWT was applied to D2 subband to extract beta (16–24 Hz) and gamma (24–32 Hz) bands. D3, D4 and A4 represent alpha (8–16 Hz), theta (4–8 Hz) and delta (0–4 Hz) band, respectively. D6, D7, D8, D9 and A9 are subbands with lower frequencies in delta band, while D1 is subband with higher frequency than gamma band. Subbands used to extract signal features are given in Table 1. The epoch energies of these subbands in each EEG channel were calculated; then, this feature vector was used as input of LSTM classifier.

### 2.2. Classification with LSTM Network

In this study, LSTM network, which is a type of RNN, was used to train a deep neural network to classify EEG data as interictal and ictal. LSTM network was created by using deep network designer in MATLAB deep learning toolbox [33]. LSTM was first introduced by Hochreiter and Schmidhuber [34]. This network was defined in [34] by Equations (1)–(6). In these equations, the summation indices u represents input units, gate units, memory cells or conventional hidden units. The *j*-th memory cell is denoted by $c_{j}$. $out_{j}$ and $in_{j}$ represent the output and input gates, respectively. Their activations at time *t* are denoted by $y^{in_{j}}\left(t\right)$ and $y^{out_{j}}\left(t\right)$, respectively. $s_{c_{j}}\left(t\right)$ represents internal state. w stands for the updating weight.

(1) $$y^{out_{j}}\left(t\right)=f_{out_{j}}\left(net_{out_{j}}\left(t\right)\right);y^{in_{j}}\left(t\right)=f_{in_{j}}\left(net_{in_{j}}\left(t\right)\right);$$

(2) $$net_{out_{j}}\left(t\right)=\sum_{u}w_{out_{ju}}y^{u}\left(t-1\right)$$

(3) $$net_{in_{j}}\left(t\right)=\sum_{u}w_{in_{ju}}y^{u}\left(t-1\right)$$

(4) $$net_{c_{j}}\left(t\right)=\sum_{u}w_{c_{ju}}y^{u}\left(t-1\right)$$

(5) $$y^{c_{j}}\left(t\right)=y^{out_{j}}\left(t\right)h\left(s_{c_{j}}\left(t\right)\right)$$

(6) $$s_{c_{j}}\left(0\right)=0;\;s_{c_{j}}\left(t\right)=s_{c_{j}}\left(t-1\right)+y^{in_{j}}\left(t\right)g\left(net_{c_{j}}\left(t\right)\right)\;for\;t>0$$

**Figure 4:**
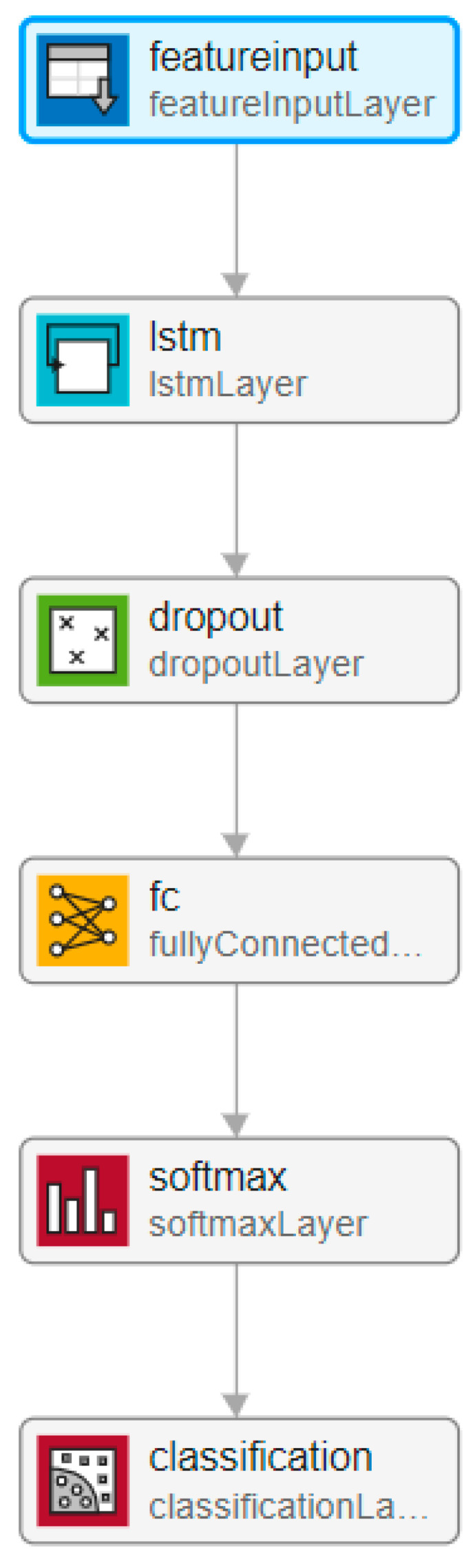
LSTM network used in the proposed method.

LSTM network generally used in analysis and classification of sequences by revealing dependencies between time steps of a sequence [35]. However, training LSTM network with raw data results in a low classification accuracy. On the other hand, classification accuracy can be increased by using features of data as an input for training process of LSTM [36]. Thus, in this study, training LSTM model was realized by using extracted features of EEG signals instead of using raw EEG data. LSTM network used in the proposed method is shown in Figure 4. 

### 2.3. Asymmetry Score Calculation

Asymmetry score was calculated from the energy ratios of these symmetrical left and right bipolar EEG channels. Asymmetry score represented by *A* is given in (7), where $x_{left}$ and $x_{right}$ represent symmetric left and right EEG channel signals of one epoch length, respectively. By using (7), asymmetry score is calculated for each symmetrical EEG channel pair listed in Table 2. Considering that there is an increase in signal energy on epileptic focus side because of excessive neural activity, if calculated asymmetry score is greater than 1, that EEG channel pair of the patient is labeled as “1”, and if asymmetry score is less than 1, it is labeled as”−1”. Then, the label numbers assigned to the eight channel pairs are summed. If the total number is greater than zero, it is determined that epileptic focus is on the left side for that EEG recording of the patient, else the epileptic focus is on the right side. Asymmetry coefficient is calculated and then labeled for each recording of each patient. If the seizure recordings labeled as left focus exceed the number of seizure recordings labeled as right focus, then patient is labeled as left TLE patient, otherwise the patient is labeled as right TLE patient.

(7) $$A=\frac{\sum x_{left}{\left(n\right)}^{2}}{\sum x_{right}{\left(n\right)}^{2}}$$

## 3. Results

In the proposed epileptic focus identification method, 76 EEG recordings of 26 TLE patients were used. As a gold standard, epileptic focuses of 48 of the 76 EEG recordings were labeled as right-sided TLE, and the epileptic focus of 28 of them was labeled as left-sided TLE by the physician. The EEG signal dataset was split into 75% training set (7452 epochs) to train deep learning algorithm, 15% validation set (1596 epochs) to tune classifier parameters and 15% testing set (1596 epochs) to evaluate the performance of the proposed algorithm. In AU dataset, the ratio of ictal epochs to interictal epochs is 926:10,652, or approximately 1:12. Data augmentation was conducted by duplicating ictal EEG signals to balance the size of ictal and interictal data. Otherwise, classifier can classify all signals as interictal to obtain high accuracy. 

Before training, the data was randomly shuffled so that the same labeled data would not be trained consecutively. Input size was set to 16 to match the feature dimension (energies of 16 channels) in feature input layer. In LSTM layer, number of hidden units was set to 128. In fully connected layer, output size was set to two, representing the number of classes. Training options were specified by assigning minimum batch size as 150, maximum epochs as 100 and initial learning rate as 0.001 and gradient threshold as two. In addition, ADAM method was selected as an optimizer. 

Results of the proposed epilepsy detection method are given in Table 3. As can be seen from this table, the highest classification accuracy was achieved by using energy feature of beta band, while the lowest one was obtained by using D9 band. Parameters of LSTM network were selected experimentally by comparing the success rates as reported in Table 4. According to this table, the highest test classification accuracy was obtained for 16-channel beta band with 150 minimum batch size. Finally, results of the proposed epileptic focus identification algorithm is given in Table 5. As can be seen in Table 5, accuracy of 96.10%, sensitivity of 100% and specificity of 93.80% were achieved in epileptic focus identification. Comparison table of proposed epileptic focus identification algorithm and related methods in the literature is given in Table 6. As seen in this comparison table, proposed algorithm obtained the highest accuracy in epileptic focus identification by comparing the existing methods [20,24].

The main purpose of this study is to identify epileptic focus by examining the asymmetries in left and right hemispheres. Success rate of 86.84% for ictal and interictal classification (Task-1), and 96.10% for epileptic focus identification (Task-2) were obtained in AU database. By applying the same algorithm, 96.67% accuracy for ictal and interictal classification (Task-1) on Bonn dataset was achieved. When compared to the studies in [16,21], a higher accuracy was achieved in the proposed study for Task-2. Accuracy of 80.00% and 96.00% for Task-2 were obtained in [20,24] on Bonn dataset, respectively, while accuracy of 96.10% was obtained in AU dataset for Task-2, which is higher than the literature results. Again, Task-1 was applied both on Bonn and AU database, and Task-2 was applied only on AU database because AU dataset was labeled according to epileptic focus besides ictal and interictal labeling by the expert. However, Bonn and CHB-MIT databases were not used in Task-2 because they do not include epileptic focus labels.

## 4. Discussion

The algorithm proposed in this study is applied on multichannel EEG recordings obtained from Ankara University hospital and Bonn database. In order to realize epilepsy classification and focus identification facts, a deep learning-based LSTM method is established and applied to multichannel EEG data to discriminate ictal periods and then to identify the region of epileptic focus. Deep learning-based algorithms have more advantages than classical classifiers like SVM, KNN, etc. Therefore, deep learning has potential for multi-class identification and may achieve more accurate results and has higher potential for handling large-scale data classification than other classical approaches [30].

All subbands of EEG signals were tested by the proposed algorithm individually. Highest classification accuracy was obtained by using energy feature of beta band (Table 3). Test classification accuracies of 79.79% and 86.84% were obtained for 18-channel and 16-channel beta band with 150 minimum batch size, respectively (Table 4). Also, epilepsy detection was realized on Bonn dataset, and 96.67% success rate was obtained. Epileptic focus identification was conducted with accuracy of 96.10%, sensitivity of 100% and specificity of 93.80% (Table 5). As can be seen from the comparison table (Table 6), while most of the studies in the literature focused on epilepsy detection (ictal and interictal classification), proposed algorithm determines epileptic focus in addition to epilepsy detection. So, results of the epileptic focus identification algorithm can only be compared with the studies cited in the references [20,24]. As can be seen in Table 6, proposed algorithm achieved the highest accuracy for epileptic focus identification by comparing the existing methods [20,24].

The epilepsy detection results given in comparison table (Table 6) were obtained by using cleared (Bonn and CHB-MIT) and also intracranial (Bonn) EEG records, so they achieved higher accuracies as expected. In fact, we also achieved high accuracy (96.67%) in ictal epoch detection task on Bonn dataset. EEG signals recorded from Ankara University hospital are raw data (without preprocessing) and has been obtained using surface electrodes. Although accuracies of epilepsy detection by using the raw EEG signals seem lower than the accuracy of the studies tested with publicly available databases, results in this study are reasonable to be applicable in clinics for treatment and surgical operation. 

The study is limited to only temporal lobe epilepsy among other epilepsy types. Future studies on epilepsy detection and focus identification from EEG recordings can be extended to other types of epilepsies, such as frontal lobe epilepsies, idiopathic generalized epilepsies, to obtain more comprehensive diagnostic medical tools. Another limitation of the proposed study may be the execution time. In current study, total execution time is 127 s, including recordings of all patients in AU database, for computer specifications mentioned in Section 2. More powerful CPU for a computer may provide fast execution close to real-time epilepsy detection and focus identification and also for real-time applications in clinics.

## Figures and Tables

**Table 1:** Subbands used in the proposed study.

Subband No	Subband Name	Frequency Range (Hz)
1	Delta	0–4
2	Theta	4–8
3	Alpha	8–16
4	Beta	16–28
5	Gamma	28–32
6	D1	32–64
7	D2	16–32
8	D5	2–4
9	D6	1–2
10	D7	0.5–1
11	D8	0.25–0.5
12	D9	0.125–0.25
13	A9	0–0.125

**Table 2:** Symmetrical bipolar EEG channels.

	Left Brain Hemisphere	Right Brain Hemisphere
1	Fp1-F7	Fp2-F8
2	F7-T3	F8-T4
3	T3-T5	T4-T6
4	T5-O1	T6-O2
5	Fp1-F3	Fp2-F4
6	F3-C3	F4-C4
7	C3-P3	C4-P4
8	P3-O1	P4-O2

**Table 3:** Ictal and interictal classification results of proposed algorithm.

Subband No	Subband Used for Energy Feature	Validation Accuracy	Test Accuracy	Training Accuracy	Execution Time
1	Delta (0–4 Hz)	0.5229	0.5520	0.5481	2 min 4 s
2	Theta (4–8 Hz)	0.8227	0.7504	0.8368	2 min 7 s
3	Alpha (8–16 Hz)	0.8029	0.8066	0.8394	2 min 6 s
4	Beta (16–28 Hz)	0.8747	0.8684	0.9415	2 min 7 s
5	Gamma (28–32 Hz)	0.8365	0.7971	0.8760	2 min 9 s
6	D1 (32–64 Hz)	0.8499	0.8499	0.9302	2 min 1 s
7	D2 (16–32 Hz)	0.8493	0.7983	0.9036	5 min 48 s
8	D5 (2–4 Hz)	0.7776	0.7979	0.8372	2 min 10 s
9	D6 (1–2 Hz)	0.6685	0.6753	0.6910	2 min 6 s
10	D7 (0.5–1 Hz)	0.6638	0.5962	0.6243	2 min 5 s
11	D8 (0.25–0.5 Hz)	0.6169	0.5694	0.6310	2 min 4 s
12	D9 (0.125–0.25 Hz)	0.4981	0.4008	0.5153	1 min 58 s
13	A9 (0–0.125 Hz)	0.5116	0.4476	0.5254	2 min 1 s

**Table 4:** Ictal and interictal classification accuracies obtained by using energy of beta band in LSTM network by changing some parameters.

Feature	Parameters	Validation Results	Test Results	Training Results	Execution Time
Energy of Beta band (16–28 Hz)	Minimum batch size = 27 Total number of channel = 18	Accuracy = 0.8656 Sensitivity = 0.7895 Specificity = 0.9417	Accuracy = 0.8239 Sensitivity = 0.7519 Specificity = 0.9328	Accuracy = 0.9187 ± 0.0593 Sensitivity = 0.9758 Specificity = 0.9522	10 min 40 s
	Minimum batch size = 27 Total number of channel = 16	Accuracy = 0.8333 Sensitivity = 0.7444 Specificity = 0.9223	Accuracy = 0.8480 Sensitivity = 0.7895 Specificity = 0.9366	Accuracy = 0.9889 ± 0.0620 Sensitivity = 0.9726 Specificity = 0.9478	11 min 8 s
	Minimum batch size = 150 Total number of channel = 18	Accuracy = 0.8769 Sensitivity = 0.8270 Specificity = 0.9267	Accuracy = 0.7979 Sensitivity = 0.7143 Specificity = 0.9242	Accuracy = 0.8992 ± 0.0408 Sensitivity = 0.9597 Specificity = 0.9525	2 min 9 s
	Minimum batch size = 150 Total number of channel = 16	Accuracy = 0.8747 Sensitivity = 0.8421 Specificity = 0.9073	Accuracy = 0.8684 Sensitivity = 0.8496 Specificity = 0.8968	Accuracy = 0.8938 ± 0.0396 Sensitivity = 0.9630 Specificity = 0.9200	2 min 7 s

**Table 5:** Results of the proposed epileptic focus identification algorithm.

Number of EEG Recordings	Result
76 (48 Right TLE, 28 Left TLE)	Accuracy = 96.10% Sensitivity = 100% Specificity = 93.80%

**Table 6:** Comparison of proposed epileptic focus identification algorithm and related methods.

Author	Dataset	Method	Task 1: Interictal and Ictal Epoch Classification	Task 2: Epileptic Focus Identification	Accuracy (%)
Türk et al. [24]	Bonn University	CNN	√	√	98.50 (Task1) 80.00 (Task2)
Daoud et al. [20]	Bern-Barcelona and Bonn	Deep convolutional autoencoder	X	√	93.21 (Bern-Barcelona) 96.00 (Bonn)
Qureshi et al. [9]	Bonn and CHB- MIT	KNN and FRNN	X	X	99.81 (Bonn) 92.79 (CHB-MIT)
Poorani et al. [18]	CHB-MIT	CNN and LSTM	√	X	94.83
Varlı et al. [19]	Bern-Barcelona, Bonn and CHB-MIT	CWT, STFT and LSTM	√	X	99.62 (Bonn)
Mir et al. [21]	CHB-MIT	LSTM	√	X	99.80
Singh et al. [22]	CHB-MIT	LSTM	√	X	98.14
Proposed method			√	√	86.84 (Task1—AU dataset) 96.67 (Task1—Bonn data) 96.10 (Task2—AU dataset)

√: The given task was performed by the related study. X: The given task was not performed by the related study.

## Data Availability

Data is not publically unavailable due to privacy and ethical restrictions.

## Nomenclature

ADAM	Adaptive Moment Estimation
ANN	Artificial Neural Network
AU	Ankara University
CHB-MIT	Children’s Hospital Boston (CHB) and the Massachusetts Institute of Technology (MIT)
CNN	Convolutional Neural Network
CWT	Continuous Wavelet Transform
DWT	Discrete Wavelet Transform
EEG	Electroencephalography
FRNN	Fuzzy Rough Nearest Neighbor
GMM	Gaussian Mixture Model
KNN	K-Nearest Neighbor
LDA	Linear Discriminant Analysis
LSTM	Long Short-Term Memory
OMP	Orthogonal Matching Pursuit
PCA	Principal Component Analysis
PNES	Psychogenic Non-Epileptic Seizure
RBF	radial basis function
ResNet	Residual Networks
RNN	Recurrent Neural Network
STFT	Short Time Fourier Transform
SVM	Support Vector Machine
TLE	Temporal Lobe Epilepsy
TUH	Temple University Hospital
USVM	Universum Support Vector Machine
WPD	Wavelet Packet Decomposition
